# Current Treatment Limitations in Age-Related Macular Degeneration and Future Approaches Based on Cell Therapy and Tissue Engineering

**DOI:** 10.1155/2014/510285

**Published:** 2014-01-14

**Authors:** P. Fernández-Robredo, A. Sancho, S. Johnen, S. Recalde, N. Gama, G. Thumann, J. Groll, A. García-Layana

**Affiliations:** ^1^Ophthalmology Experimental Laboratory, School of Medicine, University of Navarra, C/Irunlarrea 1, 31008 Pamplona, Spain; ^2^Tissue Engineering and Biomaterials Unit, CEIT and TECNUN (University of Navarra), Paseo de Manuel Lardizabal 15, 20018 San Sebastian, Spain; ^3^Department of Ophthalmology, RWTH Aachen University Hospital, Pauwelsstraße 30, 52074 Aachen, Germany; ^4^Department of Ophthalmology, Geneva University Hospitals, Faculty of Medicine, University of Geneva, Rue Alcide-Jentzer 22, 1211 Geneve 14, Switzerland; ^5^Department of Functional Materials in Medicine and Dentistry, University of Würzburg, Pleicherwall 2, 97070 Würzburg, Germany; ^6^Department of Ophthalmology, School of Medicine, Clinica Universidad de Navarra, Avenida Pio XII 36, 31008 Pamplona, Spain

## Abstract

Age-related macular degeneration (AMD) is the leading cause of blindness in the Western world. With an ageing population, it is anticipated that the number of AMD cases will increase dramatically, making a solution to this debilitating disease an urgent requirement for the socioeconomic future of the European Union and worldwide. The present paper reviews the limitations of the current therapies as well as the socioeconomic impact of the AMD. There is currently no cure available for AMD, and even palliative treatments are rare. Treatment options show several side effects, are of high cost, and only treat the consequence, not the cause of the pathology. For that reason, many options involving cell therapy mainly based on retinal and iris pigment epithelium cells as well as stem cells are being tested. Moreover, tissue engineering strategies to design and manufacture scaffolds to mimic Bruch's membrane are very diverse and under investigation. Both alternative therapies are aimed to prevent and/or cure AMD and are reviewed herein.

## 1. Age-Related Macular Degeneration: Socioeconomic Burden and Limitations of Current Therapies

### 1.1. General Introduction

Age-related macular degeneration (AMD) is one of the leading causes of vision loss and the most common cause (almost epidemic) of blindness in industrialized countries. It is the first source of legal blindness (visual acuity < 20/200) in Europe and mainly affects people over the age of 50 affecting about 30 million people worldwide. The dramatic loss of autonomy and life quality associated with AMD [1, 2] leads to increased costs for healthcare and long-term care. AMD is multifactorial but clearly age-related pathology. The number of affected people is expected to double by the year 2020 as a result of ageing of the world's population. Even, in developed countries, AMD is gaining attention due to increased life expectancy and improved visual care facilities [3].

AMD is an inflammatory chronic progressive eye disease with damage to retinal pigment epithelium (RPE) cells in its early stage, while late stage has two distinct forms: the slowly progressing “nonvascular” and the rapidly progressing “neovascular” AMD [4]. However, both forms eventually lead to blindness [5] through degeneration of retinal pigment epithelium (RPE) and posterior photoreceptor (PR) cells.

Moreover, with age, the metabolic activities of RPE cells decrease leading to deposition of debris on and in BM and decreased turnover and degradation of the extracellular matrix (ECM) resulting in altered filtration of nutrients and metabolic wastes which affects attachment of RPE cells to the BM. The resulting altered metabolism and death of the RPE cells cause the hypopigmentation observed in early AMD [6]. Probably, the presence of debris triggers a local inflammatory response that activates the immune system, causing a chronic and excessive immune response with further damage to the retina [7]. Moreover, patient and *in vitro* studies reveal that cells do not attach to old or damaged BM and do not form a monolayer over the area of degenerated RPE cells [8]. In AMD patients, BM no longer supports the normal functions of RPE cells, and the RPE cells are no longer able to maintain a normal BM [8, 9].

### 1.2. Limitations of Current Therapies in Clinical Use

Traditional therapeutic products targeting degenerative diseases have largely focused on palliative forms of treatment that mainly ameliorate or control the symptoms of a disease without addressing the underlying biological cause. There is currently no cure available for AMD, and even palliative treatments are rare. Treatment options span a broad range of therapeutic approaches, including thermal laser photocoagulation, surgical approaches (excision, displacement, or transplantation), and new treatments targeting the choroidal neovascularization (CNV) component and its pathogenic cascade, such as verteporfin with photodynamic therapy (vPDT) and more recently antivascular endothelial growth factor (VEGF) therapies [10].

#### 1.2.1. Intermediate and Advanced AMD Prevention

In addition to the intake of vitamin and minerals supplements described by the two AREDS studies, stopping smoking and a healthy diet are strongly recommended.

#### 1.2.2. Wet/Vascular AMD Treatments

*Laser Photocoagulation and Photodynamic Therapy.* Treatment strategies for the neovascular form of AMD had been focused several years ago on the prevention of further progress of the CNV either with laser photocoagulation for extrafoveal CNV [11–13] or with photodynamic therapy (PDT) [14]. Although PDT has become increasingly prevalent, its effect on the patients' vision is limited; there is a large number of CNV recurrences reported after PDT and the unpredictable repetition of treatments in 3-month intervals in PDT treatment [14–16]. Thermal laser successfully prevented the proliferation of CNV; however, visual loss and recurrences impaired the treatment benefit. Using nonthermal laser energy through vPDT appeared as a healthy alternative, but again, it was unsatisfying given the inability to improve vision in a majority of patients [10].

*Anti-VEGF Therapy.* Vascular endothelial growth factor (VEGF-A) is the most potent promoter of angiogenesis and its role in the pathogenesis of neovascular AMD is well recognized [17, 18]. The advent of intravitreous VEGF inhibitors has revolutionized the management of neovascular AMD. A portion of patients with neovascular AMD can be symptomatically treated with VEGF inhibitors that are effective in preventing the progression of vascular AMD with some vision recovery in only 30% of patients. Monthly injections of Ranibizumab or Bevacizumab (off label) are the current, gold standard therapy in the management of neovascular AMD. Individualized treatment regimens, including traditional PRN (pro-re-nata) and “treat and extend,” may yield visual outcomes comparable with monthly dosing, although close followup and frequent treatments are still needed. However, inhibition of vascularization only maintains temporarily the status quo in the majority of patients indicating that neovascularization is a late event in the development of AMD and certainly not the causative event. In addition, as VEGF is an essential factor for cell survival, it has been demonstrated that the sustained blocking of VEGF can lead to undesirable adverse effects, such as chorioretinal atrophy [19, 20]. Recent preclinical studies in monkeys clearly show that anti-VEGF therapy has a strong reducing effect on the diameter of the choriocapillaris [21]. These findings offer an explanation why RPE cells and retinal atrophy may develop.

In minimally classic/occult trial of the anti-VEGF antibody ranibizumab in the treatment of neovascular age-related macular degeneration (MARINA) study, patients receiving the 0.5 mg dose of ranibizumab experienced a 21.4 letter improvement compared with sham injections, and in ANCHOR, they demonstrated a 20.5 letter improvement compared with those receiving photodynamic therapy [22]. Data from the CATT study showed that patients who were scrutinized monthly experienced similar outcomes at years 1 and 2 whether treated monthly or PRN with ranibizumab or Bevacizumab. These results suggest that either a PRN or a treat-and-extend regimen provides a reasonable approach to the monthly injection protocol, although the two have not been compared directly with each other in prospective trials [23].

Although anti-VEGF injections have largely improve the visual outcomes of late neovascular AMD, the risk of visual decline and disease activity persists, and the need for anti-VEGF treatment continues in a substantial portion of patients. The long-time results observed after seven years in the SEVEN-UP cohort may reflect the inexorable nature of this chronic disease even in the face of treatment. In this study, 98% of the study eyes were detected to have macular atrophy which mainly involved the fovea, as indicated by definite decreased autofluorescence. Decreased visual acuity in late neovascular AMD may be associated with macular atrophy and the presence of intraretinal or subretinal fluid [20].

Anti-VEGF agents with a higher affinity for VEGF molecule, such as aflibercept (Eylea), offer another option [22]. Aflibercept is a promising new agent recently approved by FDA that may lessen the treatment burden, given the encouraging 1-year (after three initial monthly injections) results from the 2 mg bimonthly maintenance dosing arm of the phase 3 VIEW studies compared to the monthly regimen [19]. Preapproval clinical trials showed benefits and side effects that were similar to those of Ranibizumab. Similar to Ranibizumab, aflibercept binds to all VEGF isoforms (A, B, and C) with a 10-fold higher affinity than Ranibizumab for VEGF [24, 25].

*Combined Therapy.* PDT in combination with anti-VEGF and steroids is currently used as a second-line therapy in patients not responding to monotherapy with anti-VEGF agents or in whom the treatment burden of monthly injections is too great.

Combination therapy with anti-VEGF therapy and ionizing radiation offers another option to reduce treatment frequency. Radiation was never widely adopted because it did not provide a significant, reproducible effect on visual acuity, while difficulty delivering targeted doses led to complications in some patients [26–29]. New options, however, such as epimacular brachytherapy and robotic stereotactic radiotherapy, enable safer, targeted delivery of the most appropriate dosage, minimizing damage to surrounding structures and improving outcomes [30–33].

Results of the phase 3 choroidal neovascularization secondary to AMD treated with beta radiation epiretinal therapy trial, which compared epiretinal brachytherapy plus ranibizumab with ranibizumab alone, found that the combination was not noninferior to monotherapy with ranibizumab [34].

Ranibizumab efficacy as sole therapy and in combination with PDT has been evaluated in several trials and 6-month results indicate visual acuity improved by 12.8 letters with few drug-related side effects like transient inflammation ([35], Genentech press release: phase III study shows that Lucentis improved vision compared to Visudyne in patients with wet age-related macular degeneration).

#### 1.2.3. Nonvascular AMD

Currently, there are no treatments available for nonvascular AMD [36–38]. Transplantation of RPE cells alone has failed to supply a sustainably functional monolayer RPE cells, and delivery of autologous RPE cells into the subretinal space results in insufficient cell survival [39, 40]. Although there is evidence that the combination of specific vitamins might slow progression of nonexudative AMD, this involves a rather small risk group of patients. In addition, the long-term tolerability and side effects of high dose vitamin treatments must be carefully evaluated [41]. Other treatment options currently evaluated in studies are rheopheresis for extracorporeal blood filtration, which has been undertaken in patients with early stages of AMD, namely, drusen and small RPE atrophies [42–44]. Grid and focal laser application and also subthreshold laser application in patients with drusen have shown no vision recovery and an increase in the number of neovascularizations in the treated groups [15, 45, 46].

*Surgery.* Proof-of-principle for the replacement of RPE cells has been provided by several experimental surgical procedures for treating AMD [15, 47]. Macular translocation surgery is achieved by the detachment and rotation of the neural retina of the patient, to reposition the macula from the diseased macular RPE to an area of healthy RPE cells [48–50]. However, it yields only temporary recovery of vision; the large retinotomies associated with this procedure have high complication rates [51] and require further surgery to reposition extraocular muscles. Yet the procedure demonstrates two important points: (1) replacing the degenerated RPE cells does restore vision and (2) BM at the macular region is defective and cannot support normal RPE functions since, with time, the translocated RPE cells degenerate again. As translocated cells survive in their original position, RPE cells do not degenerate because of an “endogenous” defect; rather, it appears that the substratum (BM) is not appropriate for survival. The alternative was “autologous RPE transplantation,” which is accomplished by removing a small area of healthy peripheral RPE and transplanting it beneath the macula to replace the diseased RPE [52–57]. This technique carries less risk than macular translocation, but the surgical procedure is longer and showed postsurgery complications, such as retinal detachment. Retinal rotation techniques may be an alternative in a very large CNV in nonresponders to new therapies or when it is associated with large hematomas [58].

### 1.3. Socioeconomic Burden

The emotional and economic burden of AMD is often underrecognized. The prevalence of neovascular AMD (NV-AMD), which accounts for 90% of AMD-related severe visual impairment, increases exponentially with age [59, 60]. Patients with visual impairment such as AMD are more likely to have falls and fall-related injuries, lose driving privileges, experience depression, and anxiety, use special vision aids, and need assistance with day-to-day functioning [61–63], all of which are associated with higher resource utilization than the general population [64]. Moreover, the aging population will create a drain on our available healthcare resources—a burden that will continue to grow in magnitude over the next decades.

In contrast to other age-related eye diseases like cataracts that are largely solved by current therapies, the visual prognosis for most patients with AMD is poor and the late stages of both wet and dry AMD are usually associated with severe visual loss, which has profound effects on overall quality of life. The shortages in executing ordinary tasks are also extended to their psychological functioning, as evidenced by patients with AMD reporting greater emotional distress than visually intact peers [65].

Without treatment, the neovascular form of AMD often leads not only to severe loss of vision but also to considerable associated economic burden [66–68]. Despite their benefits, frequent, indefinite injections of VEGF blocking agents introduce a significant treatment burden for patients with neovascular AMD. Sequential intraocular injections of anti-VEGF agents are very expensive (1,200 euros per injection, in addition to monthly medical visits) and require its application over long periods of time (12–20 months in most cases and subsequent retreatments). This means a high cost to the National System of Health. Improvement obtained in MARINA was sustained over 2 years, remarkably, but relied on monthly injections to achieve this. This regimen is difficult for patients to maintain, particularly older patients who may not drive, and spurs investigation of other treatment options [69]. Some studies have been aimed to investigate the cost effectiveness of Ranibizumab versus Bevacizumab, given the low cost of the last one. The average 1 year cost of Bevacizumab was 50 times reduced versus Ranibizumab: US$595 in the Bevacizumab versus US$23 400 in the Ranibizumab monthly group and US$385 in the Bevacizumab versus US$13 800 in the Ranibizumab as-needed dosing group. The results of the CATT study support the use of Bevacizumab in the treatment of neovascular AMD and highlight the significant economic benefit of Bevacizumab over Ranibizumab. A recent large, multicenter, randomized prospective study (ABC Trial) which demonstrated MARINA/ANCHOR-like results lends further support for Bevacizumab use in neovascular AMD [70]. Although bimonthly injections certainly are less burdensome on patients than monthly injections, development of new pharmacologic agents and treatment modalities such as regenerative medicine are ongoing in an attempt to mitigate this burden. Interventions that improve the morbidity caused by AMD have the potential to greatly benefit the quality of life of individual patients, as well as the overall economic well-being.

The economic impact of AMD on society is expected to increase in the near future as population age and the prevalence of AMD increase. With new AMD therapies, healthcare decision makers will require reliable quantitative data on AMD-related resource utilization to evaluate alternatives, as the ones suggested in the present review.

The mean annual direct vision-related medical cost was reviewed by Cruess et al. and estimated to range from 2153€ per patient in the UK to 4390€ per patient in Canada. The mean direct nonvision related medical cost was estimated to range from 597€ (11% of the total cost) in the UK to 1657€ (21% of the total cost) in Canada. The annual societal costs estimated in 2004 for bilateral NV-AMD were €1.3 billion in Germany, €624 million in France, €511 million in the UK, €311 million in Canada, and €268 million in Spain. The mean annual cost per bilateral NV-AMD patient ranged from €5300 to 12 445, of which direct vision-related medical costs accounted for 23–63% of the total cost. These estimates are higher than those of two previous prospective studies [64]. Sharma and Oliver-Fernandez estimated the NV-AMD patient annual cost to be $Can3865 (year 2004 values, equivalent to €2715) in North America, with 90% attributable to direct medical costs (photodynamic therapy, 77%) and the remainder to nonmedical costs (home support, 6%) [71]. Similarly, a study involving two French referral centres [72] estimated a €3660 (year 2000 value) mean annual per NV-AMD patient cost, with medical costs accounting for 51% of the total.

There are extensive data that emphasize the need for new treatments for AMD that will prevent vision loss and progression to blindness in order to lessen the ensuing economic burden [73]. Overall, on the basis of current policies, age-related public expenditure is projected to increase in average by about 4.75 percentage points of GDP by 2060 in the EU and by more than 5 percentage points in the euro area—especially through pension, healthcare, and long-term care spending; all of the above concepts succeed in this pathology, and it is necessary to solve these problems in order to avoid negative effects on general European economy. Inactive people in active period of life generate an increase in pensions. Many AMD sufferers are below 65 years old and they are in active life period and they are obligated to ask for pensions. AMD is a disease related to ageing and is associated with decreased functional abilities and quality of life, which result in an increase in healthcare resource utilization.

## 2. Cell Therapy in AMD: Perspectives and Limitations

It has now been almost 40 years since Gouras and colleagues [74] showed that, using a pars plana approach, rabbit RPE cells transplanted subretinally in rabbits survived and phagocytized photoreceptor outer segments. Since Gouras' work, a number of investigators have transplanted RPE cells, iris pigment epithelial (IPE) cells, human stem cells, and genetically modified cells in a number of animal models; cell transplantation did prevent photoreceptor degradation and improved vision [39, 75–82]. The positive results of cell transplantation in animals, especially the results in the Royal College of Surgeon (RCS) rats in which RPE degeneration is followed by photoreceptor degeneration similar to AMD, led to the transplantation of cell to the subretinal space of AMD patients.

In 1997, Algvere and colleagues transplanted patches of human fetal RPE into patient with neovascular AMD as well as in patients with nonexudative AMD and concluded that “it is technically feasible to transplant human RPE into the submacular space without adversely affecting visual function in nonexudative AMD over relatively long periods of time” [83]. Since 1997, a number of investigators have transplanted RPE cells, choroid-Bruch's-RPE explants [9, 52, 53, 56, 84], and IPE cells [85–87], and recently human embryonic stem cells have been transplanted in one patient with geographic atrophy [88] and a phase I/II, open-label study to determine safety of subretinal transplantation of human embryonic stem cell [NCT01344993] in geographic atrophy (GA) patients is planned. Janssen Research and Development, LLC, plans a clinical trial (NCT01226628) that will transplant human umbilical tissue-derived cells to the subretinal space of patients with visual impairment resulting from GA.

Since the harvesting of autologous RPE cells for transplantation requires an elaborate surgical procedure, a number of investigators have transplanted IPE cells as a substitute for RPE cells since autologous IPE cells can be easily harvested [89]; *in vitro* studies have shown that RPE and IPE share many morphologic and functional similarities [85, 90, 91], and transplantation of IPE cells to the subretinal space of RCS rats prevented photoreceptor degeneration [39, 81, 92, 93]. The results of these studies have not fulfilled the promise that cell transplantation would be the solution to the treatment of AMD; cell transplantation has failed to show significant improvement in vision in AMD patients.

Even though cell transplantation has failed in the past, the successful transplantation in animal models suggests that theoretically cell transplantation has the potential to be a significant treatment for AMD. The failure of cell transplantation to improve vision in AMD patients is part of the result of the lack of knowledge of the factors that underlay development and progression of AMD. However, there is enough knowledge about AMD to be able to construct a theoretical framework necessary to eventually achieve restoration of vision by cell transplantation. In general, the approach has been to transplant autologous or homologous cell suspensions or autologous peripheral retina choroid-BM-RPE explants without taking into account the status of the patient's retina. In considering the status of the retina, two characteristics of the disease must be taken into account, namely, the severity and the factors underlying the disease. For this discussion, we will assume that the severity of the disease and loss of vision does not follow a gradient but instead is segregated into three independent phases. Specifically, initially, phase 1: RPE cells in the macular region are intact or reversibly damaged and vision loss is the result of the reversible loss of some function(s) by RPE cells and/or loss of communication between RPE cells and photoreceptors. Later, phase 2: RPE cells in the macular region are degenerated or irreversibly damaged but the photoreceptors are intact or reversibly damaged and loss of vision is the result of nonfunctional RPE cells in the macular region. Finally, phase 3: both RPE cells in the macular region and photoreceptors are degenerated or irreversibly damaged and loss of vision is due to lack of both RPE cells and photoreceptors. As for the factors underlying the disease, numerous studies have indicated that the CNV associated with neovascular AMD results from the lack of the proper balance between angiogenic (VEGF) and antiangiogenic factors (pigment epithelial derived factor, PEDF) [94, 95]. The demonstration that the overexpression of VEGF in the retina is the driving force for pathological neovascularization [96, 97] and overexpression of VEGF in the retina is responsible for neovascular AMD [98, 99] led to the development of antiangiogenic therapies for neovascular AMD. Factors underlying geographic atrophy are unknown; however, from studies on the viability and rescue of photoreceptors, it appears that factors produced by RPE cells, including basic fibroblast growth factor (bFGF), brain-derived neurotrophic factor (BDNF), and ciliary neurotrophic factor (CNTF), are responsible for normal functioning of the retina and photoreceptors' viability [100–102]. In fact, the intravitreal injection of CNTF in a feline model of hereditary retinal degeneration leads to the long-term survival of photoreceptors [103].

In the eye, RPE cells are the principal source of PEDF, a neuroprotective and cytoprotective factor and a potent inhibitor of VEGF [104, 105] that is essential in maintaining the macula avascular [106, 107]. In neovascular AMD, there is an overexpression of VEGF [18] and decreased levels of antiangiogenic factors [108–112]. Here it is interesting to note that smoking, a risk factor for neovascular AMD, is associated with poor visual improvement after anti-VEGF treatment and that nicotine increases the VEGF/PEDF ratio in RPE [113, 114] indicating that VEGF is the driving force for neovascular AMD development.

Based on the overexpression of VEGF, intravitreal administration of anti-VEGF monoclonal antibodies (Avastin or Lucentis) has become standard therapy for neovascular AMD with approximately 30% of patients gaining 3 or more lines of visual acuity and stabilization in 90% of patients [37, 115, 116]. However, the short half life of the antibodies *in vivo* requires repetitive, mostly monthly injections to maintain the therapeutic effect. In addition to the difficulty and cost of monthly treatment for elderly patients, the repetitive intravitreal injections carry substantial risks for the patient, for example, endophthalmitis, ocular hypertension, submacular haemorrhage, and retinal detachment [117, 118]. The difficulties associated with anti-VEGFs necessitate better and more lasting treatments for neovascular AMD.

It was thought that transplantation of RPE cells to the subretinal space would be a better and more permanent treatment for AMD. However, transplantation of RPE or IPE cell suspensions could not be an effective treatment for AMD since normal RPE cells would not supply the increased levels of PEDF necessary to inhibit the augmented VEGF and possibly necessary levels of growth factors required to protect photoreceptors from degenerations. An appropriate treatment for phase 1 neovascular AMD, in which RPE cells and photoreceptors are intact or reversibly damaged, would be the transplantation of genetically modified, autologous pigment epithelial (RPE or IPE) cells that would produce increased levels of VEGF inhibitor, such as PEDF and/or endostatin. In addition, the gene for the inhibitor of VEGF should be integrated into the host cell genome, such that the synthesis and secretion of the augmented PEDF would be for the life of the patient.

Cell transplantation for the treatment of phase 2 neovascular AMD, in which RPE cells are degenerated but the photoreceptors are functional, will require genetically modified, autologous, PEDF and/or endostatin-transfected pigment epithelial cells transplanted as a monolayer on a biocompatible substratum that supports RPE cell functions. A biocompatible substratum is necessary since cell suspensions transplanted to the subretinal space of AMD patients do not attach and form a monolayer [87] because the aged BM, especially neovascular AMD BM, is altered and does not appear to support good attachment and survival of pigment cells [119–123]. A number of biocompatible substrates have been investigated and have been found to support attachment, growth, and functionality of RPE cells [40, 124–126]; however, these have not been transplanted in patients. The transplanted cell monolayer would replace the degenerated cells and rejuvenate BM by synthesizing a new basal lamina.

Phase 3 of the disease, in which both RPE cells and photoreceptors are degenerated or nonfunctional, will require the manufacture and transplantation of a complex structure that encompasses a biocompatible substratum upon which autologous, PEDF, and/or endostatin transfected pigment epithelial cells are allowed to form a monolayer; the monolayer is then overlaid with photoreceptor precursor cells. In animal models of retinal degeneration, transplanted photoreceptors integrate into the host retina and improve function [127–129].

Photoreceptor precursor can be selected from iPS cells derived from the patient's fibroblasts. A number of methods have been devised to reprogram fibroblasts and induce iPS cells [130, 131]. In the case in which both RPE cells and photoreceptors are degenerated or nonfunctional, it may be necessary to transplant stem cells or iPS-derived RPE cells that may have a wider complement of factors that may be essential to reconstruct a BM-RPE photoreceptor complex [58, 132–135]. iPS derived RPE cells have been shown to form pigmented patches of typical cobble-stone cells that express the tight junction protein ZO-1, RPE65, and bestrophin and showed phagocytic activity by the uptake of fluorescent latex beads [136]. Photoreceptors have been generated from iPS cells, have been transplanted in animal models, and have been shown to integrate into the host retina and express photoreceptor markers [137, 138].

The essential goal of cell transplantation in neovascular AMD, when the RPE and photoreceptors are intact or reversibly damaged, is the effective inhibition of blood vessel invasion of the subretinal space and restoration of RPE and photoreceptor functions. For such purpose, the addition of the PEDF gene to the transplanted cells should affect blood vessel inhibition and restore RPE and photoreceptor functionality. It will be critical that the PEDF gene be integrated into the host genome so that its activity will last for the life of the patient. For such purpose, the Sleeping Beauty (SB100X) transposon system is ideal as the gene delivery system since it is highly efficient, similar to retroviral vectors, but without the associated side effects. The SB100X system delivers genetic material into a target cell genome resulting in robust and stable expression of the transgene [139–142].

For later stages of neovascular AMD and GA, it may be necessary to introduce into the cells to be transplanted not only the PEDF gene, but also other neuroprotective genes, such as CNTF and IGF1 to engender a neurogenic supportive environment. Before cell transplantation can become a routine procedure, it will be necessary to develop methodologies to identify the stage of degeneration of RPE cells and photoreceptors in AMD patients and transplant cell in an appropriate genetically modified and proper architecture.

## 3. Tissue Engineering in AMD: Materials, Scaffolds, and Material-Cell Interactions

Another challenge of cell therapy is the lack of cell engraftment and survival in the host tissue after implantation. Therefore, the need arises where this is assisted by artificial supports, known as scaffolds, which are structured biocompatible materials that mimic the host tissue. As described above, different cell types have been tested for treating AMD, including RPE, IPE, retinal progenitor cells (RPC), photoreceptor, and stem cells [143]. Previous works highlight the crucial role of cell adhesion onto BM, the despaired engraftment derived from the deterioration of this membrane, and they show that the best adhesion occurs on the RPE basal lamina [144]. Therefore, tissue engineering approaches for AMD treatment are mainly focused on the transplantation of artificial constructs that ensure cell engraftment and activity, overcoming the limitations of classical cell therapy and empowering the promises of gene therapy.

### 3.1. Material and Fabrication

The approaches from tissue engineering for the design and manufacturing of scaffolds are very diverse. Decellularized matrices are the most natural scaffolding form. The strategy consists in removing every cell from an organ or tissue and reusing it for *in vitro* recellularization and subsequent *in vivo* implantation. In the particular case of retina regeneration, different natural tissues have been tested to be used as recellularized scaffolds. In early experiments, RPE and IPE cells have been transplanted *in vitro* onto human BM from cadaveric origin [145], Descemet's membranes [146], lens capsules [147], and amniotic membranes (AM) [148, 149]. Furthermore, cell growth has been modulated by performing microcontact printing on the surface of lens capsules [150]. More recently, trabecular meshwork mesenchymal stem cells and AM have been combined, showing that differentiation towards photoreceptor-like cells is induced [151]. Despite these advances, the access to natural scaffolds is limited to donors; thus, artificial scaffolds are also being developed to elude this restriction. In soft tissues, polymeric materials are preferred for scaffold manufacture due to their tunable capacity and similarity with the host tissue in terms of mechanical properties. In the case of retina, the most used polymeric material from natural origin is collagen. Recently, *in vitro* and *in vivo* studies have been performed where RPE cells were cultured on ultrathin collagen membranes. They have proven to form cell monolayers amenable in a way that permits transplantation into subretinal space [40]. Nevertheless, the tunable capacity of the natural polymers is limited and, in some cases, they cannot provide the scaffold with the required properties; therefore, synthetic polymeric materials are chosen as a highly adjustable alternative. Some of the materials already tested for retinal regeneration are Poly(Lactic-co-Glycolic) Acid (PLGA), [152, 153], Poly(glycerol Sebacate) (PGS), [154], Polycaprolactone (PCL) [155], and Poly(methyl methacrylate) (PMMA) [156]. A detailed review of the materials investigated for retinal regeneration has been published by Hynes and Lavik [124].

Among the vast variety of techniques for the fabrication of polymeric scaffolds [157], particle leaching, phase separation, and freeze-drying are the preferred ones for porous structures. However, pointing towards mimicry of the natural ECM assembly, fibrous structures reproduce in a greater extent the microscopic morphology of the native ECM. Consequently, electrospinning has become a remarkably advantageous technique for fibrous scaffolds. With this technique, randomly oriented polymeric fibers in the range of 1 *μ*m in diameter can be deposited; yet, up to date, no precise deposition of the single fibers is possible and only the final macroscopic arrangement can be customized [158, 159]. A new technique for direct writing known as melt electrospinning has recently entered the field of polymeric scaffolds, which allows a precise control of the localization and orientation of every single electrospun fiber [160]. Nonetheless, the dimensions are still too large for a subretinal implantation and further scaling down of the features is necessary [161]. This technique, although still under development, offers new alternatives for tunable structure of polymeric scaffolds in the micrometric range. Additionally, traditional microfabrication techniques, which were originally employed in the silicon industry, are lately being explored and applied in the field of tissue engineering for the fabrication of scaffolds. Photolithography, thin film deposition, and polymer casting etcetera allow the production of well-defined microscopic topographical features on the scaffolds and enhanced cell activity [162, 163].

### 3.2. Improved Biomaterial Surfaces

Control over surface chemistry is essential to regulate the interaction between cells and scaffolds. A traditional strategy to improve cell adhesion on biomaterials has been the immobilization of proteins on the surface via incubation. Thus, collagen, fibronectin, vitronectin, laminin, and other ECM proteins were directly bound to the surface, allowing cells to anchor onto them [164]. An alternative to protein immobilization is peptide immobilization, which avoids wettability and orientation effects, and allows a tailored design of the aminoacid sequence and precise control towards cell-scaffold interaction. Peptides consist of cell recognition motifs that are found in the proteins, which integrins bind to. The best known motif is the RGD (arginine, glycine, and aspartic acid) sequence, which is prevalently present in fibronectin, although other proteins do contain varied forms of the sequence as well, such as RGDV in vitronectin or RGDT in collagen. It has been shown that cyclic RGDfK-type peptides with different conformations show enhanced integrin binding affinity and especially selectivity [165]. One of the most recent works of scaffolding as substitutes for BM is presented by Treharne et al. where they develop methacrylate-based copolymers. The copolymers are chemically modified by succinimidyl carbonate groups; thus, the hydrophilicity altered favoring peptide adhesion [166]. Results show enhanced peptide and RPE cell adhesion on succinimidyl carbonate functionalized scaffolds. Although initially surface modification was performed in synthetic scaffolds, in the last years, same strategies have started to be translated into natural origin scaffolds. An example of that is provided by Sistiabudi and colleagues where they modify the inner collagenous layer of Bruch's membrane with a collagen binding motif, where bioactive molecules are anchored [167].

Recent strategies in biomaterials development aim at the combination of increased hydrophilicity and minimized unspecific protein adsorption and cell adhesion, with the ability to permit selective cell binding by the incorporation of cell adhesion motifs. Such techniques have been developed for flat model substrates but remain challenging for biomaterials and three-dimensional structures. One promising one-step technique for the generation of nonwoven textile sheets with basal-membrane like structure and functionalization, with large potential as artificial BM, has recently been introduced [168]. The method relies on the use of an amphiphilic macromolecular additive based on star-shaped PEO to clinically used biodegradable polyesters during fibre generation by electrospinning. This way, hydrophilic fibres are obtained, on which protein adsorption and cell adhesion are minimized. However, cell adhesion peptides can be immobilized on the surface of these fibres, so that specific adhesion of cells onto the fibrous sheets results. With this method, scaffolds could be produced that influence the reaction of immune cells [169] and that mimic the basal membrane in skin [170]. Hence, such strategies bear great potential for TE approaches in AMD, where materials that mimic the BM are one key factor for success.
